# Use of medical administrative data for the surveillance of psychotic disorders in France

**DOI:** 10.1186/s12888-017-1555-0

**Published:** 2017-12-04

**Authors:** Christine Chan Chee, Francis Chin, Catherine Ha, Nathalie Beltzer, Christophe Bonaldi

**Affiliations:** Direction of Non Communicable Diseases and Trauma, French National Public Health Agency, 12, rue du Val d’Osne, 94415 Saint-Maurice cedex, France

**Keywords:** Psychotic disorders, Medical administrative databases, Methodology, France, Prevalence

## Abstract

**Background:**

Psychotic disorders are among the most severe psychiatric disorders that have great effects on the individuals and the society. For surveillance of chronic low prevalence conditions such as psychotic disorders, medical administrative databases can be useful due to their large coverage of the population, their continuous availability and low costs with possibility of linkage between different databases. The aims of this study are to identify the population with psychotic disorders by different algorithms based on the French medical administrative data and examine the prevalence and characteristics of this population in 2014.

**Methods:**

The health insurance system covers the entire population living in France and all reimbursements of ambulatory care in private practice are included in a national health insurance claim database, which can be linked with the national hospital discharge databases. Three algorithms were used to select most appropriately persons with psychotic disorders through data from hospital discharge databases, reimbursements for psychotropic medication and full insurance coverage for chronic and costly conditions.

**Results:**

In France in 2014, estimates of the number of individuals with psychotic disorders were 469,587 (54.6% males) including 237,808 with schizophrenia (63.6% males). Of those, 77.0% with psychotic disorders and 70.8% with schizophrenia received exclusively ambulatory care. Prevalence rates of psychotic disorders were 7.4 per 1000 inhabitants (8.3 in males and 6.4 in females) and 3.8 per 1000 inhabitants (4.9 in males and 2.6 in females) for schizophrenia. Prevalence of psychotic disorders reached a maximum of 14 per 1000 in males between 35 and 49 years old then decreased with age while in females, the highest rate of 10 per 1000 was reached at age 50 without decrease with advancing age. No such plateau was observed in schizophrenia.

**Discussion:**

This study is the first in France using an exhaustive sample of medical administrative data to derive prevalence rates for psychotic disorders. Although only individuals in contact with healthcare services were included, the rates were congruent with reported estimates from systematic reviews. The feasibility of this study will allow the implementation of a national surveillance of psychotic disorders essential for healthcare management and policy planning.

## Background

Mental disorders are common worldwide and are leading causes of years lived with disability [1]. One of the challenges for every country is to establish its own burden of mental conditions and estimate the socio-economic costs involved [2]. The implementation of a comprehensive national surveillance system specific to mental disorders is necessary for measuring, monitoring and subsequently improving prevention, resource allocation and mental healthcare policy [3]. Different methods for mental health surveillance exist, mostly depending on the information needed and the resources available. Prevalence rates for common mental disorders are usually derived from population-based epidemiological surveys [4]. However for chronic low prevalence disorders such as psychotic disorders, epidemiological surveys may not be the best approach because of limitations in population sample size, inadequate screening instruments and bias due to selective non-participation in community surveys [5]. Psychotic disorders including schizophrenia, characterized by delusions and hallucinations are among the most severe psychiatric disorders that have a great effect on the individuals affected and on the society with long-term symptoms, disability, unemployment and reduced life expectancy [6]. On the other hand, most people living with psychotic disorders have contact with health services and hence the use of administrative data may be particularly helpful for developing epidemiological studies on these severe mental disorders [7]. Medical administrative databases are increasingly used for epidemiological projects in developed countries due to their large coverage of the population, their continuous availability and low costs with possibility of linkage between different databases at an individual level over several years. Although the use of administrative datasets rarely allows access to the patients’ medical records and investigations based on these datasets rely on the diagnoses recorded [3], a few studies including one carried out in France that compared psychiatric diagnoses in administrative datasets with clinical diagnoses recorded in the patients’ charts consistently found a high level of agreement for schizophrenia [8–10]. In the only French study, Richieri et al. found a Kappa coefficient of 0.8 for schizophrenia while comparing the diagnoses of 112 patients reported in administrative databases and those established by an independent psychiatrist after a thorough interview of the patients [10].

However, due to deinstitutionalization of mental health care, up to 25% of the patients with schizophrenia receive exclusively outpatient care and identifying patients with schizophrenia or psychotic disorders should not rely only on hospitalization based data [11].

In France, health care is provided in both public settings and private practices. The health insurance system covers the entire population of 66 million living in France including the overseas territories and is divided in different schemes based on professional status. The claims for reimbursement of ambulatory care for all the health insurance schemes are centralized in a unique national database, the national inter-schemes Health Insurance Information system (S*ystème National d’Information Interrégimes de l’Assurance Maladie, SNIIRAM*). Interestingly, this database can be linked at an individual level with the national hospital discharge databases.

Santé publique France, the French Public Health Agency, has the missions of epidemiological surveillance and monitoring of the population health status, and the development of prevention, health promotion and health education. In order to fulfill this surveillance task, access to the SNIIRAM and the hospital discharge databases is legally allowed to the French Public Health Agency.^1^

The aims of this study are to identify the population with psychotic disorders by different algorithms based on the French medical administrative data and examine the prevalence and characteristics of this population in 2014. This study is the first step of a larger project implementing a national surveillance of psychotic disorders in France with trends over time, regional patterns, comorbidities and mortality.

## Methods

### Databases

The SNIIRAM database comprises more than 1.2 billion detailed claims for the reimbursement of ambulatory care in private practice (medical consultations and procedures, biological tests, medication…) without indication on the related diagnosis [12]. However, there are 30 chronic severe and costly affections (*affections de longue durée, ALD*) including chronic psychiatric conditions for which the eligible patient’s ALD-related care is free of charge. Request for inscription of a patient on the ALD affection list is made by the general practitioner and approved by a medical officer from the health insurance scheme [13].

The national hospital discharge databases collect all admissions in every public or private hospital including general hospitals (*programme de médicalisation des systèmes d’information en médecine, chirurgie, obstétrique, PMSI-MCO*) and psychiatric hospitals (*recueil d’informations médicalisé en psychiatrie, RIM-P*). In addition, the RIM-P also gathers information on outpatient psychiatric care in the public services. While the PMSI-MCO has been functional for at least 20 years, the RIM-P has only been introduced nationally in 2006. Completeness and quality of the RIM-P data have been evaluated as correct for epidemiological studies as from 2010 [14].

Linkage of the SNIIRAM and hospital discharge databases is performed through a unique national identification number for each patient, except for RIM-P outpatient care in the public psychiatric services. The latter can only be linked with the RIM-P hospitalization in psychiatric wards via a different specific identification number present in the RIM-P.

In both the SNIIRAM and hospital discharge databases, the patient’s sociodemographic characteristics are available, such as sex, age and place of residence. In addition, in the SNIIRAM system, there is a proxy variable for social deprivation (beneficiaries of the universal complementary health insurance, *CMUC* granted to a person with low income^2^).

### Algorithms for inclusion of individuals with psychotic disorders

Psychotropic medication is coded according to the Anatomical Therapeutic Chemical (ATC) classification index. As antipsychotics are not specific for psychotic disorders and can be prescribed for other conditions, we considered for inclusion patients who had at least one hospital-based diagnosis of psychotic disorder in the past 4 years (2010 through 2013) and at least three claims of antipsychotics (ATC code N05A excluding Lithium) at different dates during the studied year 2014.

In the ALD and hospital discharge databases including public outpatient psychiatric care, the diagnoses are coded according to ICD-10. In the hospital discharge databases, diagnoses are separated in principal diagnosis (PD) defined as the main reason for admission and associated diagnoses (AD) defined as conditions which significantly influenced healthcare during the hospital stay. For psychotic disorders, we considered for inclusion individuals with ICD-10 codes F20 to F29 and for schizophrenia those with code F20.

In order to determine as accurately as possible the number of individuals with psychotic disorders in 2014, we examined three algorithms with patients on ALD and/or antipsychotic medication and included successively hospitalization with only a PD of psychotic disorders; hospitalization with psychotic disorders as PD or AD; and outpatient care or hospitalization with psychotic disorders as PD or AD. The details of the algorithms for psychotic disorders were as follows:

- Algorithm 1: Patients were included if they had at least one of the following: (a) a psychotic disorder code in the ALD database in 2014; (b) at least 3 claims for antipsychotic medication in 2014 with at least one hospitalization in the past 4 years with a discharge diagnosis of psychotic disorder; (c) at least one hospitalization in 2014 with a PD of psychotic disorder in the PMSI-MCO database (general hospitals) or in the RIM-P database (psychiatric hospitals).
- Algorithm 2: same as algorithm 1 for (a) ALD and (b) medication, but for (c) either PD or AD of psychotic disorder in the PMSI-MCO or RIM-P databases.
- Algorithm 3: same as algorithm 2 and in addition, outpatient care was considered: (d) diagnosis of psychotic disorder (PD or AD) from the public outpatient psychiatric settings in 2014. Outpatient psychiatric data was linked with the inpatient psychiatric data for the years 2010 to 2013 in order to retrieve the specific identification number only present in the RIM-P database. A patient can thus be included only if there has been at least one hospitalization in psychiatry during the previous 4 years followed by outpatient care in 2014.

### Analysis

Prevalence rates were calculated by sex, age and region. Numerators were number of individuals identified by the algorithms and denominator population were mid-year census estimates provided by the National Institute of Statistics and Economic Studies. For comparison between deprived and non-deprived population, only individuals under the age of 60 were taken into account since the income threshold for eligibility for CMUC is lower than the minimal social welfare allowances for people aged 60 or older [15]. Prevalence rates by sex and region were standardized by the method of direct standardization using the age structure of the European Union 2011–2030 population projections [16]. Statistical analyses were performed with SAS Enterprise Guide 7.1 software.

## Results

Results from the three algorithms are shown in Table 1. The number of individuals with psychotic disorders rose from 446,848 for algorithm 1 to 469,587 for algorithm 3 with the inclusion of associated diagnoses and ambulatory care. Those with schizophrenia went from 224,748 to 237,808. The proportion of males was 54.6% with psychotic disorders and 63.6% with schizophrenia.

**Table 1:** Number of individuals with psychotic disorders and schizophrenia in 2014 according to the three algorithms

	Algorithm 1		Algorithm 2		Algorithm 3	
ALD	2014		2014		2014	
Hospitalization	2014 (principal diagnosis)		2014 (principal or associated diagnosis)		2014 (principal or associated diagnosis)	
Medication	N05A in 2014 + hospitalization 2010–2013		N05A in 2014 + hospitalization 2010–2013		N05A in 2014 + hospitalization 2010–2013	
Psychiatric outpatient care in public settings	–		–		Psychiatric ambulatory care 2014 + hospitalization in psychiatry 2010–2013	
Psychotic disorders						
Males	244,942	54.8%	249,796	54.6%	256,480	54.6%
Females	201,906	45.2%	207,444	45.5%	213,107	45.5%
Total	446,848	100.0%	457,240	100.0%	469,587	100.0%
Schizophrenia						
Males	143,286	63.8%	145,685	63.6%	150,953	63.6%
Females	81,462	36.2%	83,315	36.4%	86,855	36.4%
Total	224,748	100.0%	229,000	100.0%	237,808	100.0%

Considering our case definition following the fullest algorithm, Table 2 shows the contribution of each database to the diagnosis of psychotic disorders or schizophrenia. For both diagnoses, the most important contribution came from ALD: 71.9% of the individuals (73.5% in men and 69.9% in women) with psychotic disorders and 64.6% with schizophrenia (66.0% in men and 62.1% in women) benefited from ALD. One person in five with psychotic disorders and one in four with schizophrenia have been hospitalized in psychiatry in 2014 and only 10% or less in general hospitals. According to our case definition for psychotic disorders, 27.1% of the patients have been hospitalized in the past 4 years with a discharge diagnosis of psychotic disorders and were still in outpatient psychiatric care in 2014; and 35.7% have been hospitalized in the past 4 years with a discharge diagnosis of psychotic disorders and were still on antipsychotic medication in 2014. For schizophrenia, the rates were respectively 33.6% for outpatient care and 40.7% for medication. *In fine*, in 2014, 77.0% of the patients with psychotic disorders and 70.8% of those with schizophrenia received exclusively ambulatory care without any hospitalization during the year.

**Table 2:** Contribution of each database to the diagnosis of psychotic disorders or schizophrenia by sex

	Psychotic disorders			Schizophrenia		
Databases	Males *N* = 256,480	Females *N* = 213,107	Both *N* = 469,587	Males *N* = 150,953	Females *N* = 86,855	Both *N* = 237,808
	(%)	(%)	(%)	(%)	(%)	(%)
ALD	73.5	69.9	71.9	66.0	62.1	64.6
Antipsychotic medication	36.6	34.7	35.7	41.6	39.4	40.7
Public outpatient psychiatric care	29.5	24.2	27.1	35.2	31.0	33.6
Hospitalization in psychiatry	23.2	18.0	20.8	26.9	22.0	25.1
Hospitalization in general hospitals	9.6	10.5	10.0	6.6	6.2	6.5

### Prevalence rates of psychotic disorders and schizophrenia

Considering the different algorithms, prevalence rates ranged between 7.0 and 7.4 per 1000 inhabitants for psychotic disorders and between 3.5 and 3.8 per 1000 inhabitants for schizophrenia. Since the prevalence rates were very close, only rates yielded by the fullest algorithm will be presented thereafter.

Prevalence rates of psychotic disorders in 2014 in France were 7.4 per 1000 (8.3 in males and 6.4 in females), and 3.8 per 1000 (4.9 in males and 2.6 in females) for schizophrenia. The prevalence of psychotic disorders in the deprived population beneficiary of the CMUC was higher than that of the rest of the population, respectively 10.9 per 1000 (15.2 in males and 7.7 in females) vs. 6.6 per 1000 (8.2 in males and 5.0 in females). For schizophrenia, the prevalence in the CMUC beneficiaries was 5.8 per 1000 (8.9 in males and 3.6 in females) while in non-beneficiaries of the CMUC, the prevalence was 3.8 per 1000 (5.2 in males and 2.5 in females).

**Fig. 1:**
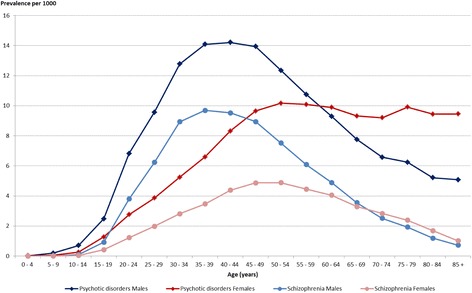
Prevalence rates of psychotic disorders and schizophrenia by age and sex in France, 2014

The persons identified by the algorithm were mostly young and middle aged: 79% of the men and 66% of the women with psychotic disorders, and 85% of the men and 78% of the women with schizophrenia were between 25 and 65 years old. Before the age of 10, no psychotic disorder was noted. Prevalence of psychotic disorders reached a maximum of 14 per 1000 in males between 35 and 49 years old then decreased steadily with age reaching a prevalence of 5 per 1000 at age 80 while in females, the highest rate of 10 per 1000 was reached 10 years later, at age 50 without showing any decrease with advancing age (Fig. 1). The trend in the male prevalence rates after age 50 was a result of a decrease in the rates of all the indicators used for the algorithm except hospitalization in general hospitals which remained at the same level. In women, rates of the indicators based on care in psychiatric settings (hospitalization and outpatient) declined with advancing age while those based on ALD and on medication remained stable until the age group 75–79 and hospitalization rates in general hospital increased.

For schizophrenia, the trend in the prevalence rates in men followed the same distribution as for psychotic disorders with a maximum of 9.7 at age 35–39 while in women, the highest rate was 4.9 per 1000 at age 45–54 and decreased with age reaching 1 per 1000 in the oldest olds. The trend in the prevalence rates for elderly women did not show the same plateau as for psychotic disorders.

**Fig. 2:**
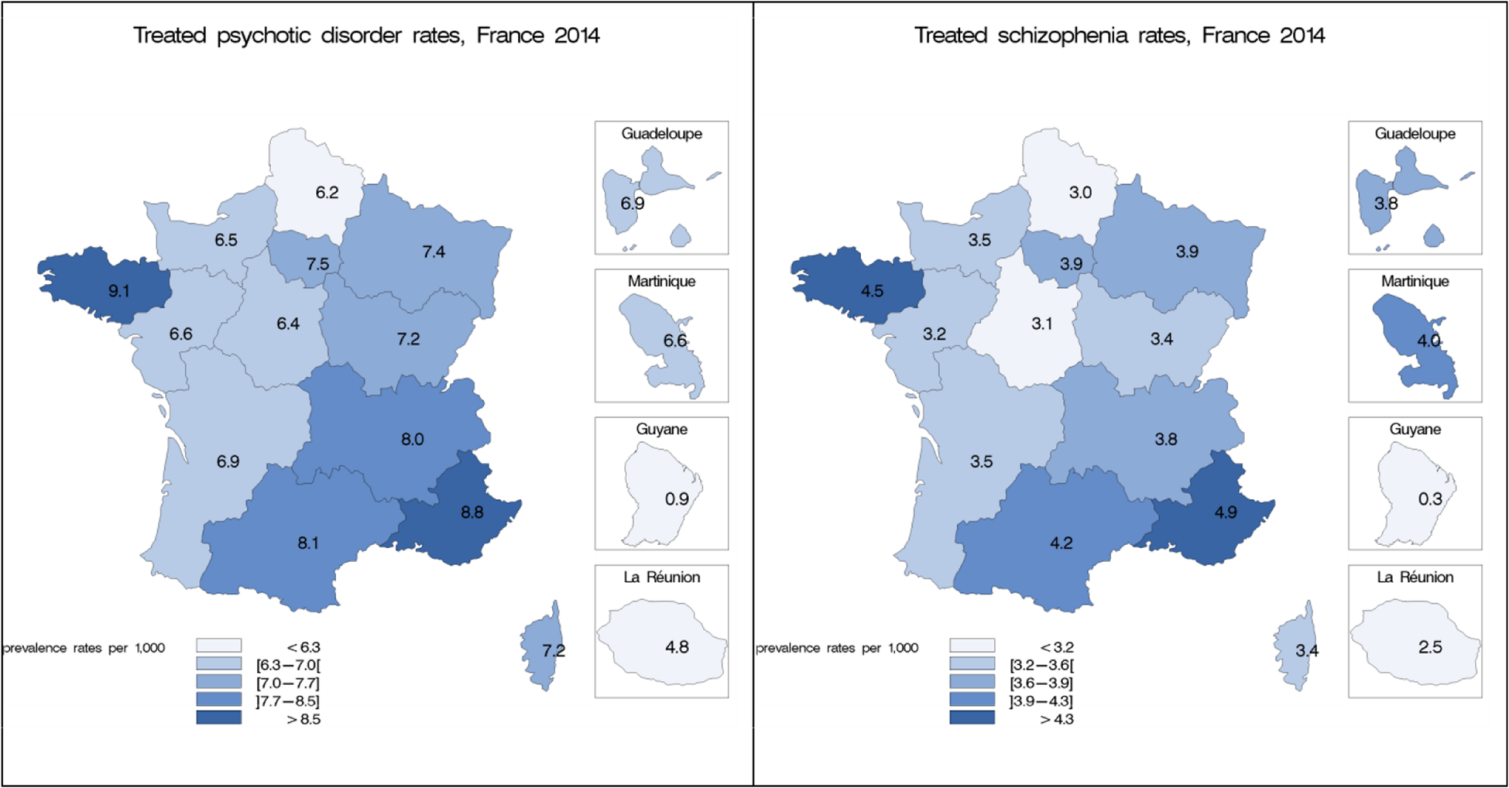
Prevalence rates of psychotic disorders and schizophrenia by region in France, 2014

Figure 2 shows the differences in the regional distribution. Rates for psychotic disorders went from 0.9 to 9.1 per 1000 and rates for schizophrenia from 0.3 to 4.9 per 1000. Brittany in the north-west and Provence-Alpes-Côte d’Azur in the south-east presented the highest regional rates (more than 15% above the national rate) while the overseas departments of French Guiana in South America and Reunion Island in the Indian Ocean showed the lowest rates. However, considering metropolitan France, the differences in the regional prevalence rates were less important with a 1.5-fold variation.

## Discussion

To the best of our knowledge, very few studies investigating the prevalence of psychotic disorders have been led in France, some of these in the specific population of male inmates [17] and the homeless [18, 19] with very high prevalence rates. In the general population, only four studies to date have taken place in different areas (very limited for most of them) at different periods using different methods. In 1970, Brunetti found a 1-year prevalence of 12 per 1000 for psychotic disorders in a rural village of 680 inhabitants in the south of France [20] and in the 1990’s, Jay and colleagues found a 1-year prevalence of 7.5 per 1000 for schizophrenia in the French overseas Reunion Island [21]. The largest study, the French Mental Health in General Population Survey interviewed 37,000 adults in 47 sites between 1999 and 2003 with the Mini International Neuropsychiatric Interview which is a standardized questionnaire more appropriate for common psychiatric disorders than for psychotic disorders [22]. With this method, estimates of lifetime prevalence rates for psychotic disorders were as high as 27 per 1000 (7 per 1000 for single psychotic episode and 20 per 1000 for recurrent psychotic episodes) [23]. The last and most recent study conducted in a suburban city near Paris in 2014 gave an estimated prevalence of 3.7 per 1000 persons with psychotic disorders in contact with public or private medical practitioners in the catchment area [24]. As such, data on prevalence rates for schizophrenia and psychotic disorders are scarce in France and show very high variation in the rates.

Our estimates of prevalence rates for psychotic disorders and schizophrenia were derived for the first time for the entire population living in France from administrative data. The case definition included individuals with full insurance coverage for these specific long-term and costly affections, and/or those hospitalized during the year with these diagnoses, and/or those with a history of hospitalization for these conditions and still on antipsychotics, or still on outpatient care in public psychiatric services with these diagnoses. The three algorithms tested took into account variations in the diagnoses (principal or associated) and hospitalization in psychiatry followed by outpatient care. For surveillance purposes, the algorithm using associated diagnoses was preferred because, according to coding instructions, associated diagnoses are noted in the hospitalization discharge databases when these conditions constituted a significant burden during the hospital stay, meaning that persisting symptoms were probably still active.

Following our case definitions, 71.9% of the patients with psychotic disorders and 64.6% of those with schizophrenia had a full insurance coverage through the ALD list system. These high percentages reflect the fact that inscription of patients with chronic psychotic disorders on the ALD list by their GP is recommended and very common in the French medical practice to ensure complete reimbursement of the patients’ medical expenditures related to their conditions. Only about one person in five with psychotic disorder has been hospitalized during the year showing that people with psychotic disorders are mainly on ambulatory care. Consequently, patients having been hospitalized in the past 4 years and still on outpatient care or on medication contributed respectively for only 27.1% and 35.7% of the case definition for psychosis. Considering that antipsychotic medication are not specific for psychotic disorders, we preferred to be conservative in our case definition and decided to link antipsychotic medication to a diagnosis of psychotic disorder from a precedent hospitalization. Thereby, we could have excluded from our algorithm psychotic patients who were only on medication without any hospitalization during the past 4 years, for example those stabilized by their medication with exclusive ambulatory care in private practices by the GP or the specialist, but we hypothesized that most of these patients would be on ALD and therefore included in our case definition.

Eventually the prevalence rates found by our algorithm were 7.4 per 1000 persons for psychotic disorders and 3.8 per 1000 for schizophrenia. Although these prevalence rates considered only patients in contact with the health care system and may underestimate the “real” prevalence, our results are congruent with those presented in three systematic reviews published to date. In the most recent review based on 21 studies of 12-month prevalence of schizophrenia in the general population, Simeone and colleagues found a median estimate of 3.3 per 1000 persons (interquartile rates: 2.6–5.1) across all studies with a median estimate of 3.1 per 1000 in European countries and 5.1 per 1000 in North America [25]. The largest review by Saha and colleagues based on 42 estimates of period (1–12 months) prevalence rates of schizophrenia in the general population also concluded that the median prevalence rate was 3.3 per 1000 persons and the 10%–90% quantiles ranged from 1.3 to 8.2 [26]. In both reviews, the median value for prevalence rates was preferred as being more appropriate than the arithmetical mean to assess central tendency for skewed distribution. The important variations of the rates presented in the reviews were due to differences in study design, dates or geographic location, and in case definition and identification. The last review by Goldner and colleagues was based on community surveys and identified 18 studies that provided a pooled estimate of 1-year prevalence of 6.0 per 1000 for psychotic disorders and 3.4 per 1000 for schizophrenia [27].

Regarding prevalence estimates according to gender, our male: female ratio of 1.9 for schizophrenia was not indicated in the review by Saha and colleagues who found no significant difference in the prevalence estimates for men and women, respectively 3.6 per 1000 in women and 3.8 in men, with a median male: female ratio of 1.1 (10% - 90% quantile range: 0.5–1.7) but the authors concluded that sex ratio of prevalence estimates may vary between sites [26]. However, in some recent studies including Szoke’s study in the Parisian area, higher rates were found in men than in women (relative risk = 1.7) [24]. Our male: female ratio for psychotic disorders yielded a lower estimate of 1.3, in accordance with the literature that sex differences depend on the stringency of the diagnosis criteria: the broader the criteria, the greater the proportion of women compared to men diagnosed with psychotic disorders. These sex differences express the interaction of differences in presentation (more negative symptoms in men vs. more mood and atypical psychotic symptoms in women), course of illness (better prognosis in women), treatment response and neurodevelopmental processes [28, 29].

One of the advantages of using data based on the whole population is the possibility of performing analyses by sex and age. Thus we have been able to show differences in the trends of psychotic disorders rates according to age in men and women. First, there is a delay of 10 to 15 years between the highest rates in males and females for both psychotic disorders and schizophrenia, highest rates in men being in the 35–49 age group and at age 50 in women. Whether this gap is due to a later onset of the disorder in women or a greater delay in the diagnosis or in the treatment is not clear and has yet to be explored. An earlier onset in males compared to females by 3 to 5 years is usually reported and can be partly attributed to a higher proportion of men with psychotic disorders who have a history of illicit substance abuse [30]. We also found that the prevalence rates for psychotic disorders in men decreased after age 50 while in females a plateau was maintained with advancing age. This differential trend according to sex and age was not found in patients with the specific diagnosis of schizophrenia: the prevalence rates decreased with advancing age in males and females. Several reasons can account for the decrease in the prevalence rates with advancing age, either a decrease in the number of patients in the healthcare system or a decrease in the absolute number of patients due to complete recovery but most probably due to a higher mortality and a lower mean age of death of individuals with psychosis and schizophrenia compared to the general population [31]. In addition, our algorithm may perhaps be less appropriate for including aged patients with psychotic disorders, hence underestimating this population. However, this decrease with advancing age was not found in women with psychotic disorders. In the elderly women, we found a stability in the rates of attribution of full health insurance coverage (ALD) and of antipsychotic treatment which counterbalanced the decrease in the rates of psychiatric hospitalization and ambulatory care. This result suggests that most probably, late onset of psychotic-like symptoms and delusions in ageing women manageable in ambulatory private practice without need for specific psychiatric care conduct their GP to prescribe antipsychotics and ask for their inscription on the ALD list because of the significant financial burden of the disease. Thus, these incident cases of less severe and less specific symptoms can account for the stability of the prevalence rates of psychotic disorders in ageing women.

We also found evidence of higher prevalence rates in the socioeconomic deprived population than in the rest of the population. Association between deprivation indices and high level of psychotic prevalence rates has been reported in several studies [32–34]. The processes by which socioeconomic levels are related to mental illness can either be selection before and during the prodromal phase of the disorder, or social drift of the mentally ill persons after onset of the disorder. Although the social causation-selection debate is still not entirely resolved [34], in the case of psychosis, it is most probable that people drift into lower social classes because of discrimination or educational and occupational disabilities [32, 33].

The major strength of this study is the use of an exhaustive sample of the population who were in contact with healthcare services for psychotic disorders. Even if we derived our estimates for 1 year, we used linked data from different databases and several years. There are some limitations in our study that have to be acknowledged. First, using administrative data do not account for cases that are not in the health care system. Furthermore, the probability being in the health system depends on several factors, such as the burden of the condition, the availability of services, their location and accessibility. However, for severe conditions such as psychotic disorders, administrative data can be an interesting approach because most of the persons get in contact with the healthcare system at some point. Therefore, our study was based on multiple data sources accounting for the array of services and interventions offered by the French healthcare system thereby increasing the likelihood of identifying prevalent cases and improving the sensitivity of our case definition. Although we tried to be as exhaustive as possible, some cases can still be missed, e.g. patients in ambulatory care in the private sector without attribution of full health insurance coverage and without hospitalization in the past 4 years. Second, the use of administrative data does not allow any verification of the diagnosis or of the actual clinical status of the patient. We acknowledge that diagnostic accuracy is important for surveillance purposes and we used information derived from health records with diagnoses based on comprehensive clinical assessment. Although diagnoses can vary among the practitioners, high levels of agreement are found for the diagnosis of schizophrenia in France [10]. Third, the number of sociodemographic variables included in the administrative databases were limited. Information about marital status, education, employment or occupation are not available although they are important factors for social support and functioning.

## Conclusion

Our study has demonstrated the feasibility of using routinely collected administrative data to derive population-based estimates of prevalence rates of schizophrenia and psychotic disorders congruent with reported estimates from systematic reviews. These results are very encouraging for Santé publique France in her perspective of implementing a national surveillance of psychotic disorders in France. The coverage of the whole population and the continuous availability of data at very low costs make it now possible to develop this surveillance with trends over time (annual prevalence rates), comparison of regional patterns, comorbidities and mortality associated with psychotic disorders. This surveillance is essential for healthcare management and policy planning with the aim of improving accessibility to medical care and reducing social and territorial inequalities. In addition, these administrative data also provide an infrastructural basis for cohort studies of longitudinal access to health services or drop out from services of patients with psychotic disorders, and for epidemiological surveys for supplementary information about their family history, premorbid history, social integration, social support and functioning.

## Abbreviations

AD: Associated diagnosis
ALD: Chronic severe and costly affections;
ATC: Anatomical therapeutic chemical
CMUC: Universal complementary health insurance;
PD: Principal diagnosis;
PMSI-MCO: National discharge databases for general hospitals;
RIM-P: National discharge databases for psychiatric hospitals;
SNIIRAM: National inter-schemes Health Insurance Information system.

## Footnotes

1
https://www.legifrance.gouv.fr/affichTexte.do?cidTexte=JORFTEXT000027830713

2 For 2017, the annual income limit for CMUC is 8723 euros for a single person.
